# Sustainability and nutritional composition of food choices in hospital canteens: a pre-post intervention study

**DOI:** 10.3389/fnut.2026.1939620

**Published:** 2026-09-11

**Authors:** Elisa Mansutti, Federica Fiori, Diana Menis, Peter Cautero, Caterina Liudmila Graziani, Daniela Zago, Marco Driutti, Lucia Lesa, Lucrezia Grillone, Francesca Cortelazzo, Angelica Cosolo, Manuela Mauro, Giulia Carioni, Enrico Scarpis, Alessandro Conte, Maria Parpinel, Laura Brunelli

**Affiliations:** 1 Department of Medicine, University of Udine, Udine, Italy; 2 Health District of Gemona, Friuli Centrale Healthcare University Trust, Gemona, Italy; 3 Medical Directorate, Hospital “Santa Maria della Misericordia” of Udine, Friuli Centrale Healthcare University Trust, Udine, Italy; 4 Health District of Udine, Friuli Centrale Healthcare University Trust, Udine, Italy; 5 Institute of Hygiene and Evaluative Epidemiology, Friuli Centrale Healthcare and University Trust, Udine, Italy; 6 Food Hygiene and Nutrition Unit, Department of Prevention, Friuli Centrale Healthcare University Trust, Udine, Italy; 7 Regional Health Coordination Agency (ARCS), Udine, Italy; 8 Division of Epidemiology and Biostatistics, IEO European Institute of Oncology IRCCS, Milan, Italy; 9 Medical Directorate, Hospital of Latisana-Palmanova, Friuli Centrale Healthcare University Trust, Palmanova, Italy; 10 Medical Directorate, Hospital of San Daniele-Tolmezzo, Friuli Centrale Healthcare University Trust, San Daniele, Italy; 11 Accreditation, Quality, and Clinical Risk Unit, Friuli Centrale Healthcare University Trust, Udine, Italy

**Keywords:** educational intervention, environmental intervention, food choices, nutritional composition, sustainability, workplace canteen

## Abstract

**Background:**

Hospital canteens provide an effective setting to improve users' dietary habits. The study evaluates users' food choices—considering nutritional composition and environmental impact -after an educational and environmental intervention, comparing the results with pre-intervention choices.

**Methods:**

A pre-post intervention study was conducted in three hospital canteens (C1, C2, C3) in northeastern Italy, during two index weeks in September 2022 (T0) and 2023 (T1). Canteen users were recruited before and after an intervention consisting of posters on healthy eating, descriptive norm messages, and environmental changes regarding fruit and vegetables. Photos of participants' lunch trays were collected, and food choices were analyzed for nutritional composition and sustainability. Descriptive statistics and appropriate non-parametric and chi-square tests were used to compare outcomes between assessment periods and across canteens.

**Results:**

2,851 trays were analyzed: 1,227 at T0 (798 in C1, 228 in C2 and 201 in C3) and 1,624 at T1 (1,005 in C1, 348 in C2, 271 in C3). In C1 and C3, there was an increase in median energy (+30 kcal; +135 kcal) compared to pre-intervention meals, while in C2 there was a decrease (−118 kcal). Despite a slight improvement in macronutrient composition, at T1 meals in all canteens were still high in lipids (30%E; 39%E; 35%E) and low in carbohydrates (44%E; 39%E; 41%E). The fiber value fell within the reference range only in C1 and C3. The median carbon (CF) and water (WF) footprints of meals in all canteens remained high: at T1 CF ranged from 966 g CO_2_eq. to 1,227 g CO_2_eq. and WF from 1,025 L H_2_O to 1,207 L H_2_O.

**Conclusion:**

The intervention has led to partial improvements in food choices. To achieve more significant results, it may be necessary to implement a parallel intervention on food offer.

## 1 Introduction

Given the predicted 20% increase in demand for land-based animal products by 2050 (1), the need to change eating habits is becoming increasingly urgent for both human and environmental health. The global aim is to gradually shift dietary habits, particularly by reducing red meat consumption, especially from ruminants, and encouraging the adoption of plant-based alternatives (2), which offer the greatest nutritional and environmental benefits (3). In this context, public health policies that inform and guide consumers toward healthier and more sustainable diets are essential. While political action is crucial, it must be complemented by educational initiatives to inform people about the health and environmental effects of their diet. Consumers play a key role, as their choices significantly influence those of others, both through direct social interactions and via social networks (4), and drive the market. In this context, canteens provide a particularly favorable environment for targeted interventions, as they are settings where an individual's food choices can influence colleagues and the impact extends to many users. Consequently, an educational intervention in canteens may also have positive effects on those who have not directly benefitted from the intervention (5). Nonetheless, changing deeply ingrained eating habits is one of the most complex challenges in public health. Several effective approaches in modifying food choices in the food service sector have been identified in the literature, including:

a) Environmental changes: these involve altering the physical or social context independently of users, including modifications to the physical environment, infrastructure, processes, procedures, and social engagement (6). Examples include informative labeling of each dish, changes to food offerings (e.g., increasing the availability of healthy options), strategic food placement, pricing policies, and contextual regulations (e.g., prioritizing plant-based options in self-service). Environmental changes also include prompting, i.e., interventions in the physical environment (e.g., decorating spaces with references to plant-based foods) and enhancing the sensory and visual appeal of foods, including new preparation methods (7–9);
b) Knowledge and behavioral regulation: this includes interventions based on the dissemination of information through educational resources (posters/flyers, courses), the development of skills, and the use of incentives such as discounts, gadgets, or verbal prompts by canteen staff to encourage the desired behavior (10);
c) Descriptive norm messages: messages based on social norms, as a nudging strategy, exploit the influence of the reference group to guide individual choices, correcting misperceptions about the behaviors of others (11, 12). Some authors suggest that descriptive norm messages can promote healthier and more sustainable food choices (13, 14), although findings are still mixed, with some studies reporting limited or no significant effects (15);
d) Rewardings: these refer to the use of material (gadgets), financial, or verbal rewards following the achievement of specific behavioral goals (10).

Furthermore, food choices may also be indirectly modified by interventions in the food offer. Indeed, recent interventions in hospital cafeterias inspired by dietary models such as the Planetary Health Diet have demonstrated that increasing the availability of plant-based foods and reducing meat can improve the nutritional quality of meals, promote healthier choices, and reduce the environmental impact of food service (16). Studies by Jaworowska (17) and Stern (18) highlight that food choices in hospitals are often nutritionally suboptimal and associated with a high environmental impact, indicating considerable scope for improvement. Hospital canteens or cafeterias may serve as an optimal setting for the implementation of healthy and sustainable food policies that protect both human and environmental health, as hospitals are not only places of patient care but also major meal providers for employees. From the sustainability perspective, food services play a key role because they can generate environmental impacts across the entire food supply chain, from procurement to food preparation, consumption and waste management (19). Indeed, recent reviews have highlighted that hospitals are strategic settings for promoting environmentally sustainable food systems. These reviews also indicate that, although several strategies to improve the sustainability of hospital food services have been proposed, particularly on food waste reduction, further studies evaluating their environmental impacts and effectiveness are needed (19, 20). From the nutritional perspective, to our knowledge, limited data specific on hospital staff canteen settings are present in the literature. Jaworowska (17) found that the vegetarian and meat-based lunches in hospital staff canteens were high in energy, salt, and fats, and that most lunch meals analyzed were characterized by an unfavorable nutrient profile. Similar evidence, with a tendency of high fat meals, was also found in a previous work by the same research group as this study (21).

The aim of this study was to evaluate users' choices regarding their nutritional composition and environmental impact of their meals after an educational and environmental intervention at the Friuli Centrale Healthcare University Trust (northeast Italy), and to compare the results with those from before the intervention.

## 2 Materials and methods

This pre-post intervention study is part of a larger project aimed at improving the nutritional and environmental awareness of hospital users, resulting from collaboration between the Department of Medicine at the University of Udine (Italy), catering service managers, the management of the Friuli Centrale Healthcare University Trust, and the local network of Health Promoting Hospitals and Health Services (HPH&HS). The results of the initial assessment of users' food choices have already been published (21). This paper presents the results of a second assessment, conducted after an intervention aimed at promoting healthier and more sustainable food choices in the same hospital canteens. The methodology for data collection and analysis was consistent with that previously described (21).

### 2.1 Data collection

The study was conducted in three hospital canteens in the province of Udine: Udine (C1), Palmanova (C2), and San Daniele (C3). The canteens differ in terms of management—external for C1 and C2, internal for C3—and daily attendance (approximately 450–480 users in C1, 100–120 in C2, and 90 in C3). The study population included hospital employees who used the canteen service on weekdays in September 2023, the same month investigated in the first survey (September 2022) to limit possible seasonal effects. Employees were allowed to select a combination of different courses for their meal according to Italian eating tradition, including a first course, second course, side dish (i.e. cooked vegetables) or salad, bread or substitutes, grated cheese, and dessert or fruit. As in the pre-intervention assessment, trays were marked with a unique code and photographed. During lunch, participants provided written informed consent and completed a questionnaire linked to the same code. The questionnaire collected socio-demographic and work-related data. Participants whose questionnaires were returned empty were excluded from the analysis.

### 2.2 Intervention

The educational intervention comprised various elements, combined in a personalized manner for each canteen according to the specific organization (type of catering management, internal vs. external) and the structural possibilities of the spaces. For example, the recommended tray composition from a nutritional and sustainable perspective, created by the dietitians of the local Department of Prevention and presented as poster displayed in the canteen, was feasible only in the canteens with an external catering service and a rotational menu. In the internally managed canteen (C3), the menu was decided daily, making the recommended menu intervention inapplicable. The food offering was not modified as part of the intervention at this stage of the project. However, despite the assessment being conducted during the same period of the year and under the same canteen management conditions as the first evaluation, the dishes offered were not identical due to normal menu variability, particularly in C3, were due to the internal management of the canteen, the menu was not predetermined.

The elements of the intervention were as follows:

a) Educational element: different types of posters were displayed for 2 months before the week of analysis (T1) in the canteen spaces.
    i Posters of the Mediterranean Diet Pyramid and Healthy Eating Plate: graphic representations of the Mediterranean Diet as a sustainable food model (22) and the Healthy Eating Plate created by the Department of Nutrition, Harvard T.H. Chan School of Public Health (23). The number of posters varied for each canteen depending on the size of the room.
   ii Posters with QR codes for further information on the food pyramid and ultraprocessed foods: these posters were in A4 format, one per canteen, displayed in a location easily visible to all canteen users.
  iii Poster with the recommended composition of the tray (only possible in C1 and C2 as mentioned above): a daily poster with the recommended tray composition from a nutritional and sustainable perspective. The poster included the recommended composition for each day of the week. The posters were in A2 format and were changed weekly by the canteen staff.
b) Nudging with environmental restructuring element. This was implemented about 2 months before T1 and included:
    i Reversing the flow of dish choices: instead of starting with the choice of first and second courses, users were encouraged to begin with salad and side dishes before proceeding to other meal components. This intervention aimed to nudge the choice of vegetables over other food categories (10) and was introduced only in C2, where this structural change was possible.
   ii Fruit, salad, and pulses self-service: in C1, salads, fruits, and pulses, instead of being served by canteen staff, were made available for self-service. Users could then decide their own portion. However, the limit of one fruit per user still applied.
c) Nudging with descriptive norm messages, based on the results of T0 and displayed as posters. For each canteen, the researchers calculated the percentages of users at T0 who chose fresh fruit as a dessert, who chose at least one portion of vegetables and, only for C1 where soft drinks were available, the percentage of users who chose water instead of soft drinks. One poster per descriptive norm message per canteen was displayed as close as possible to the relevant item. All descriptive norm message posters were displayed for about 2 months before the week of analysis. At the beginning of the survey week, these posters were removed to assess users' knowledge gained after their exposure during the previous weeks.

In summary, the interventions for each canteen were as follows:

C1: educational posters; fruit, pulses, and salad self-service; posters with descriptive norm messages.
C2: educational posters; reversing the flow of dish choice; posters with descriptive norm messages.
C3: educational posters (except the suggested composition of the tray); posters with descriptive norm messages.

### 2.3 Study outcomes

The recipes and standard portion sizes for each dish offered during the observation period were provided by the canteen staff. This information was matched at the ingredient level (24), with the foods in the Italian Food Composition Database for Epidemiological Studies (25) and of the SU-EATABLE LIFE dataset (26) to obtain the energy and nutrient composition, as well as the environmental indicators (i.e., carbon footprint—CF; water footprint—WF) for each portion of each offered dish. Due to the lack of post marketing data in the SU-EATABLE LIFE dataset, the environmental analysis did not include downstream life-cycle stages, such as food preparation (cooking) and transport, that in our case occurred in C1 and C2 for some recipes that were moved from the cooking center to the canteen. Therefore, the absolute carbon and water footprint values reported here should be considered conservative estimates. For the analysis of food choices, we considered all the dishes included in each tray. As a second step, the analysis of the photos of the meal was performed. The researchers were trained to identify the standard portion for each food item by viewing a sample of photographs with different portion sizes. Users' choices were visually estimated independently by two researchers to identify recipes and estimate portions compared to the standard portion (e.g., 50%, 100%, 150%). Discrepancies were resolved through discussion between the two researchers and a third researcher. By combining the data extracted from the photos with the recipe and food composition database, the researchers estimated the energy, nutritional profile, and environmental impact for each individual tray. Finally, the researchers compared the median values of CF and WF for each canteen with the reference ranges (CF: 800–1,000 g CO_2_ equivalents/meal, WF: 700–1,000 L H_2_0/meal) of the SU-EATABLE LIFE project (27), while the median nutrient values were compared with the Italian DRVs (28).

Finally, users' choices were analyzed based on their trays and dishes categories (vegan, vegetarian, non-beef meat, fish, beef). The trays were divided into the above categories as follows: “beef” if the tray contained at least one dish with beef; “fish” if the tray contained at least one fish dish; “non-beef meat” if there was at least one dish with meat excluding beef; “vegetarian” if there were no meat or fish dishes; “vegan” if there was no food source of animal origin on the tray.

### 2.4 Statistical analysis

A descriptive analysis was conducted on participants' characteristics associated with each tray, as well as users' choices expressed in terms of energy and nutrient composition and the carbon and water footprint of the trays. Categorical variables were presented as absolute numbers and percentages. Age class was categorized in I ≤ 34 years, II 35–54 years, III ≥55 years. Nutritional and environmental variables were not normally distributed according to the Shapiro-Wilk test and were therefore reported as medians and 25th-75th percentiles. The Mann-Whitney U test was used to compare quantitative variables between T0 and T1 within each canteen. Differences in median values of nutritional and environmental variables across canteens were assessed using the Kruskal–Wallis test; when significant, *post hoc* comparisons were performed using Dunn's test with Bonferroni adjustment. A *p*-value < 0.05 was considered statistically significant. Tray and dish categories (vegan, vegetarian, non-beef meat, fish, beef) were described using absolute and percentage frequencies. The chi-square test was used to assess associations between tray category and assessment time (T0 vs. T1), and Pearson's standardized residuals were examined. Similarly, the frequency of main-course choices within each category was calculated for each canteen, including a “no choice” option when no main course was selected. The chi-square test was also applied to evaluate associations between having a side dish and assessment time (T0 vs. T1), and between having fruit and assessment time (T0 vs. T1). Data were analyzed with STATA (StataCorp. 2021. Stata Statistical Software: Release 17. College Station, TX: StataCorp LLC) and JASP Team (2024). JASP (Version 0.19.3) [Computer software].

All data were analyzed in aggregate form to ensure that the identity of individual participants could not be traced. The study was approved by the Institutional Review Board of the University of Udine (Italy) on 06/07/2022. All participants gave informed consent to take part in the study, and all procedures complied with the ethical standards set out in the Declaration of Helsinki.

## 3 Results

### 3.1 Characteristics of the study sample

A total of 1,624 lunch meals were analyzed at T1 (1,005 from C1, 348 from C2, 271 from C3). In all canteens, participants were predominantly female (52% in C1, 58% in C2 and 63% in C3) and the mean age of participants was higher in C3 (48 ± 10 years) than in C2 (46 ± 11 years) and C1 (42 ± 12 years). Regarding professional profile, physicians was the most represented category in C1 (27%) and C3 (30%), while technician was the most represented in C2 (28%). The majority of participants were non-shift workers. A full description of the sample at T1, compared with those at T0, can be found in Table 1.

**Table 1:** Participant characteristics associated to each tray analyzed between T0 and T1, for each canteen.

User characteristics	C1		C2		C3	
	T0 (*n* = 798)	T1 (*n* = 1,005)	T0 (*n* = 228)	T1 (*n* = 348)	T0 (*n* = 201)	T1 (*n* = 271)
Age						
≤34 years	239 (30)	358 (35.6)	71 (31.1)	76 (21.8)	26 (12.9)	37 (13.7)
35–54 years	382 (47.9)	467 (46.5)	107 (46.9)	183 (52.6)	109 (54.9)	153 (56.5)
≥55 years	177 (22.2)	180 (17.9)	50 (21.9)	89 (25.6)	66 (32.8)	81 (29.9)
Missing	0 (0)	0 (0)	0 (0)	0 (0)	0 (0)	0 (0)
Sex						
Female	402 (50.4)	526 (52.3)	150 (65.8)	201 (57.8)	140 (69.7)	172 (63.5)
Male	396 (49.6)	479 (47.7)	78 (34.2)	147 (42.29)	61 (30.4)	99 (36.5)
Missing	0 (0)	0 (0)	0 (0)	0 (0)	0 (0)	0 (0)
Professional profile						
Physicians	187 (23.4)	268 (26.7)	18 (7.9)	32 (9.2)	30 (14.9)	82 (30.3)
Nurse	96 (12)	137 (13.6)	62 (27.2)	82 (23.6)	47 (23.4)	46 (17)
Health care assistant	22 (2.8)	63 (6.3)	24 (10.5)	24 (6.9)	15 (7.5)	25 (9.2)
Administrative staff	174 (21.8)	188 (18.7)	44 (19.3)	39 (11.2)	43 (21.4)	35 (12.9)
Technician	209 (26.2)	135 (13.4)	46 (20.2)	99 (28.5)	21 (10.5)	27 (10)
Others	102 (12.8)	206 (20.5)	34 (14.9)	72 (20.7)	45 (22.4)	47 (17.3)
Missing	8 (1.0)	8 (0.8)	0 (0)	0 (0)	0 (0)	9 (3.3)
Type of work						
Shift worker	202 (25.3)	285 (28.4)	49 (21.5)	88 (25.3)	36 (17.9)	85 (31.4)
Non-shift worker	552 (69.2)	660 (65.7)	163 (71.5)	255 (73.3)	161 (80.1)	164 (60.5)
Missing	44 (5.5)	60 (6)	16 (7.0)	5 (1.4)	4 (2.0)	22 (8.1)

The values are presented as absolute frequencies and column percentages. C1, canteen 1; C2, canteen 2; C3, canteen 3.

Samples at T0 and T1 were comparable in terms of sex distribution across all canteens however, only in C3 there was no difference in age distribution, with the most represented age group being 35–54 years (54.2% at T0 and 56.5% at T1). In C1, although there was difference between the two assessments, the most represented age group was 35–54 years in both T0 and T1 (47.9% and 46.5% respectively), while the least represented was ≥55 years (22.2% and 17.9% respectively). In C2, the most represented age group was 35–54 years (46.9% and 52.6% respectively), while the least represented was ≥55 years at T0 (21.9%) and ≤ 34 years at T1 (21.8%). The type of work distribution was similar between T0 and T1 in C1 and C2; in C3, non-shift workers increased by 13.5%. Professional profile distribution was different between the two assessments in all three canteens.

### 3.2 Nutritional values and sustainability of food choices in the post-intervention phase

The energy, nutrient content and the environmental impact of the trays at T1 differed between the three canteens (*p* < 0.05), except for saturated fatty acids (SFA) (see Table 2). In particular, the median energy content of the trays was higher in C1 (884 kcal/tray) compared to C2 (782 kcal/tray) and C3 (781 kcal/tray). This difference was significant in the Dunn test between C1 and C2, and between C1 and C3 (Supplementary Table 1). Most differences in macronutrients were observed between C1 and C2 (*p* < 0.001), except for SFA and monounsaturated fatty acids (MUFA) content. The highest carbohydrate and protein contents were found in C1 (carbohydrates: 104.4 g/tray, 45%E; proteins: 43.2 g/tray, 20%E), while the highest lipid content was found in C2 (33.2 g/tray, 39%E).

**Table 2:** Energy, nutrients content and sustainability indicators of the choices in C1, C2 and C3 at T1 expressed as medians, 25^th^ and 75^th^ percentiles.

Variables	C1 (*n* = 1,005)	C2 (*n* = 348)	C3 (*n* = 271)	*p*-value^a^
**Energy (kcal)**	884 [705–1,083]	782 [624–925]	781 [615–935]	0.0001
**Protein (%E)**	20 [15–25]	19 [15–23]	20 [17–24]	0.0189
**Lipids (%E)**	30 [25–38]	39 [34–46]	35 [31–41]	0.0001
SFA (%E)	7 [5–9]	8 [7–10]	9 [6–10]	0.0001
MUFA (%E)	15 [12–19]	18 [15–23]	19 [16–23]	0.0001
PUFA (%E)	5 [3–6]	7 [5–9]	5 [3–8]	0.0001
**Carbohydrates (%E)**	45 [36–53]	39 [32–46]	41 [34–46]	0.0001
Sugars (%E)	11 [8–16]	9 [6–13]	11 [9–15]	0.0001
**Fiber (%E)**	3 [2–3]	2 [2–3]	3 [2–4]	0.0001
**Protein (g/tray)**	43.2 [31.4–57.3]	34.5 [28.1–44.4]	38.2 [29.4–49.1]	0.0001
**Lipids (g/tray)**	28.9 [22.2–38.9]	33.2 [24.8–42.9]	30.4 [23.9–37.5]	0.0001
SFA (g/tray)	6.5 [4.6–10.0]	6.7 [4.8–9.9]	7.1 [4.8–10.1]	0.6026
MUFA (g/tray)	14.7 [11.5–18.6]	15.0 [11.4–19.9]	16.0 [12.6–20.7]	0.0120
PUFA (g/tray)	4.5 [3.1–6.3]	5.7 [4.2–8.1]	3.7 [2.9–6.8]	0.0001
EPA + DHA (mg/tray)	58 [0–252]	25 [0–116]	30 [0–95]	0.0001
**Carbohydrates (g/tray)**	104.4 [76.3–141.7]	79.7 [58.5–103.0]	80.7 [64.1–105.6]	0.0001
Sugars (g/tray)	26.7 [17.8–38.8]	18.3 [11.2–26.3]	24.0 [18.8–30.2]	0.0001
**Fiber (g/tray)**	11.1 [7.6–15.0]	7.8 [5.5–11.7]	11.3 [8.6–15.3]	0.0001
**CF (g CO**_**2**_ **eq./tray)**	1,227 [897–1,756]	966 [638–1,284]	1,126 [775–1,585]	0.0001
**WF (L H**_**2**_ **O/tray)**	1,207 [952–1,557]	1,025 [767–1,311]	1,195 [881–1,641]	0.0001

^a^Kruskal-Wallis test,

From a sustainability perspective (Table 2), the median values of CF and WF were higher in C1 (CF 1,227 g CO_2_eq./tray; WF 1,207 L H_2_O/tray) than in C3 (CF 1,126 g CO_2_eq./tray; WF 1,195 L H_2_O/tray) and C2 (CF 966 g CO_2_eq./tray; WF 1,025 L H_2_O/tray). This difference was statistically significant (*p* < 0.026) between all canteens (Table 3).

**Table 3:** Trays containing fruit, side dish and salad at T0 and T1, reported as absolute and relative frequencies.

Food category	C1				C2				C3			
Fruit	T0	T1	Total	*p-*value^b^	T0	T1	Total	*p-*value^b^	T0	T1	Total	*p-*value^b^
				*p* = 0.062				*p* = 0.855				*p* = 0.644
**Yes**	456 (57.1)	530 (52.7)	986		102 (44.7)	153 (44)	255		155 (77.1)	204 (75.3)	359	
**No**	342 (42.9)	475 (47.3)	817		126 (55.3)	195 (56)	321		46 (22.9)	67 (24.7)	113	
**Total (** ***n*** **)**	798	1,005	1,803		228	348	576		201	271	472	
**Side dish**	**T0**	**T1**	**Total**	***p-*** **value** ^b^	**T0**	**T1**	**Total**	***p-*** **value** ^b^	**T0**	**T1**	**Total**	***p-*** **value** ^b^
				*p* = 0.020				*p* = 0.980				*p* = 0.011
**Yes**	531 (66.5)	720 (71.6)	1,251		128 (56.1)	195 (56)	323		128 (63.7)	202 (74.5)	330	
**No**	267 (25.6)	285 (28.4)	552		100 (43.9)	153 (44)	253		73 (36.3)	69 (25.5)	142	
**Total (** ***n*** **)**	798	1,005	1,803		228	348	576		201	271	472	
**Salad**	**T0**	**T1**	**Total**	***p-*** **value** ^b^	**T0**	**T1**	**Total**	***p-*** **value** ^b^	**T0**	**T1**	**Total**	***p-*** **value** ^b^
				*p* < 0.001				*p* = 0.049				*p* = 0.034
**Yes**	186 (23.3)	401 (39.9)	587		116 (50.9)	206 (59.2)	322		66 (32.8)	115 (42.4)	181	
**No**	612 (76.7)	604 (60.1)	1,216		112 (49.1)	142 (40.8)	254		135 (67.2)	156 (57.6)	291	
**Total (** ***n*** **)**	798	1,005	1,803		228	348	576		201	271	472	

The values are presented as absolute frequencies and column percentages; ^b^Chi-squared test.

As the intervention was implemented in different combinations across the three canteens, and the canteens themselves are not comparable in terms of food offer, management type, and user rates, we analyzed each canteen separately.

**Figure 1:**
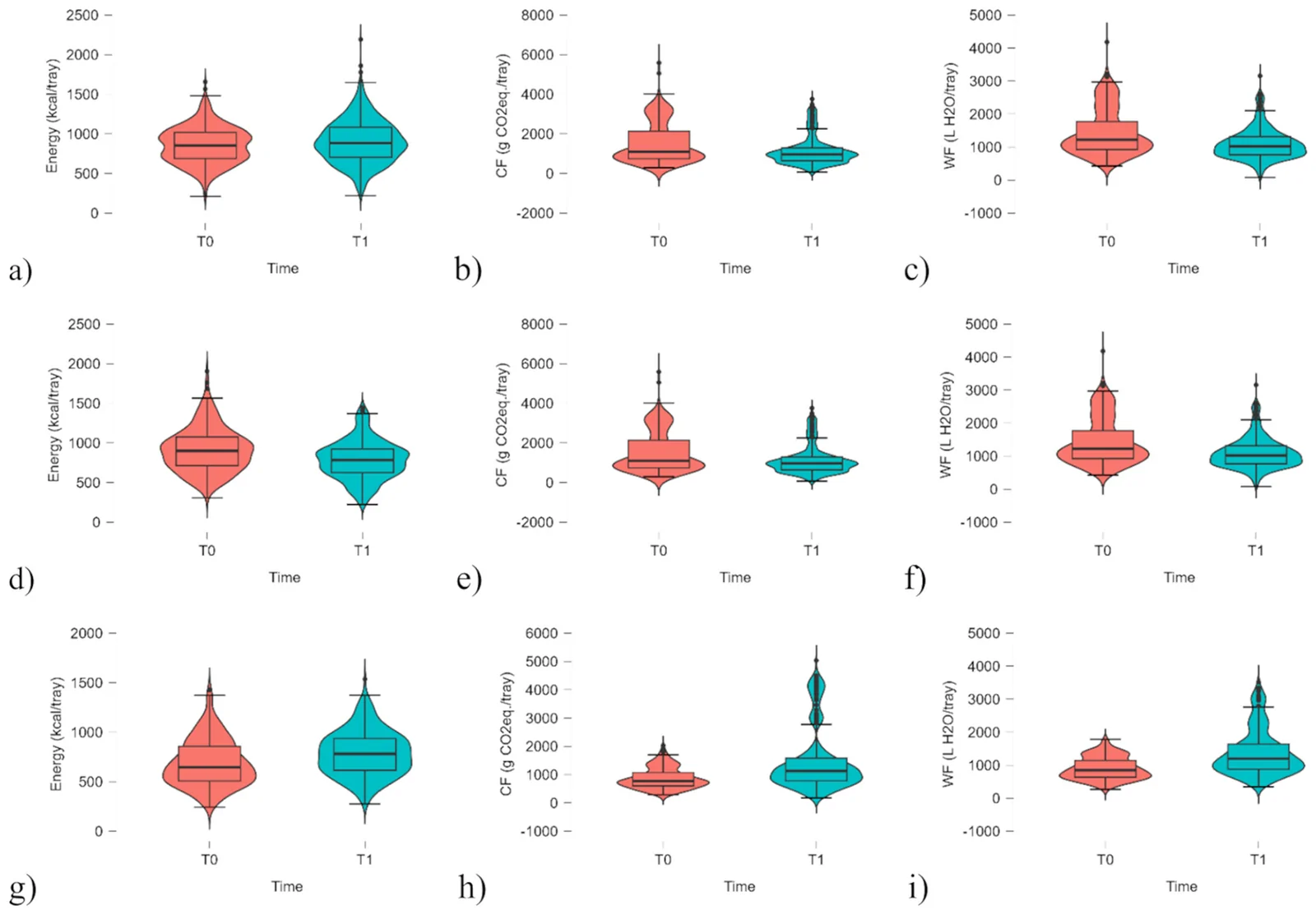
Distribution of energy values, CF and WF for C1 (letters a, b and c), C2 (letters d, e and f) and C3 (letters g, h and i) at T0 and T1.

Figure 1 shows that in C1, the median energy intake of the meal increased in the post-intervention measurement compared to T0 (854 kcal at T0 vs. 884 kcal at T1; *p* < 0.001). Considering the content in grams per tray, carbohydrates (103 g at T0 vs. 104.4 g at T1; *p* < 0.002) and fiber (9.9 g at T0 vs. 11.1 g at T1; *p* < 0.001) increased (Supplementary Table 2), while protein (45.7 g at T0 vs. 43.2 g at T1; *p* = 0.083) and lipids (28.9 g at T0 vs. 29.9 g at T1; *p* = 0.743) remained unchanged. As the energy content of the meal increased, the energy contribution of carbohydrates did not increase significantly (44%E at T0 vs. 45%E at T1; *p* = 0.639), while the energy from protein (21%E at T0 vs. 20%E at T1; *p* < 0.001) and lipids (32%E at T0 vs. 30%E at T1; *p* = 0.002) decreased. Similarly, although the content of both SFA and MUFA in grams increased in the meal at T1, only the energy contribution of MUFA increased, while that of SFA decreased (*p* < 0.001). Regarding sustainability, CF decreased significantly between T0 and T1 (1,338 g CO_2_ eq./meal at T0 vs. 1,227 g CO_2_ eq./meal at T1; *p* < 0.001), despite the total energy of the meal increased (Figure 1). The change in WF, however, was not significant (*p* = 0.107).

In C2, the median energy intake decreased (900 kcal at T0 vs. 782 kcal at T1; *p* < 0.001), accompanied by a decrease in grams of carbohydrates (93.1 g at T0 vs. 79.7 g at T1; *p* < 0.001), sugars (25.1 g at T0 vs. 18.3 g at T1; *p* < 0.001), protein (37.5 g at T0 vs. 34.5 g at T1; *p* = 0.030), lipids (37.7 g at T0 vs. 33.2 g at T1; *p* < 0.001), and fiber (10.1 g at T0 vs. 7.8 g at T1; *p* < 0.001). Only long-chain omega-3 fatty acids (EPA+DHA) increased (*p* < 0.021). In terms of energy contribution, carbohydrates remained unchanged (39%E at T0 vs. 39%E at T1; *p* = 0.265), as did lipids (40%E at T0 vs. 39%E at T1; *p* = 0.806), while the contribution of protein increased (17%E at T0 vs. 19%E at T1; *p* < 0.001). There was a decrease in the contribution of SFAs (*p* = 0.009), an increase in that of polyunsaturated fatty acids (PUFAs) (*p* < 0.001), while MUFAs remained unchanged (*p* = 0.067). In parallel to the decrease in meal energy (Figure 1), both CF and WF decreased (CF: 1,094 g CO_2_ eq./meal at T0 vs. 966 g CO_2_ eq./meal at T1; WF: 1,220 at T0 vs. 1,025 at T1; p < 0.001).

In C3, energy intake increased (646 kcal at T0 vs. 781 kcal at T1; *p* < 0.001). There was an increase in grams of protein (32.4 g at T0 vs. 38.2 g at T1; *p* < 0.001), total lipids (25.4 g at T0 vs. 30.4 g at T1; *p* < 0.001), MUFA and PUFA, and fiber (8.5 g at T0 vs. 11.3 g at T1; *p* < 0.001). There was also an increase in the energy contribution of lipids (34%E at T0 vs. 35%E at T1; *p* = 0.004) and a decrease in the contribution of carbohydrates (44%E at T0 vs. 41%E at T1; *p* < 0.001). For the energy contribution of fatty acids, there was a decrease in SFA (*p* = 0.015) and an increase in MUFA and PUFA (*p* < 0.001 and *p* = 0.016, respectively). Parallel to energy (Figure 1), CF and WF both increased (CF: 773 g CO_2_ eq./meal at T0 vs. 1,126 g CO_2_ eq./meal at T1; WF: 847 at T0 vs. 1,195 at T1; *p* < 0.001). The comparison of nutritional values and sustainability of food choices between T0 and T1 in the three canteens (C1, C2, and C3) can be found in Supplementary Table 2.

The full charts showing the distribution of nutritional and sustainability values are available in Supplementary Figures 1–3 for C1, C2 and C3 respectively.

### 3.3 Food choices

**Figure 2:**
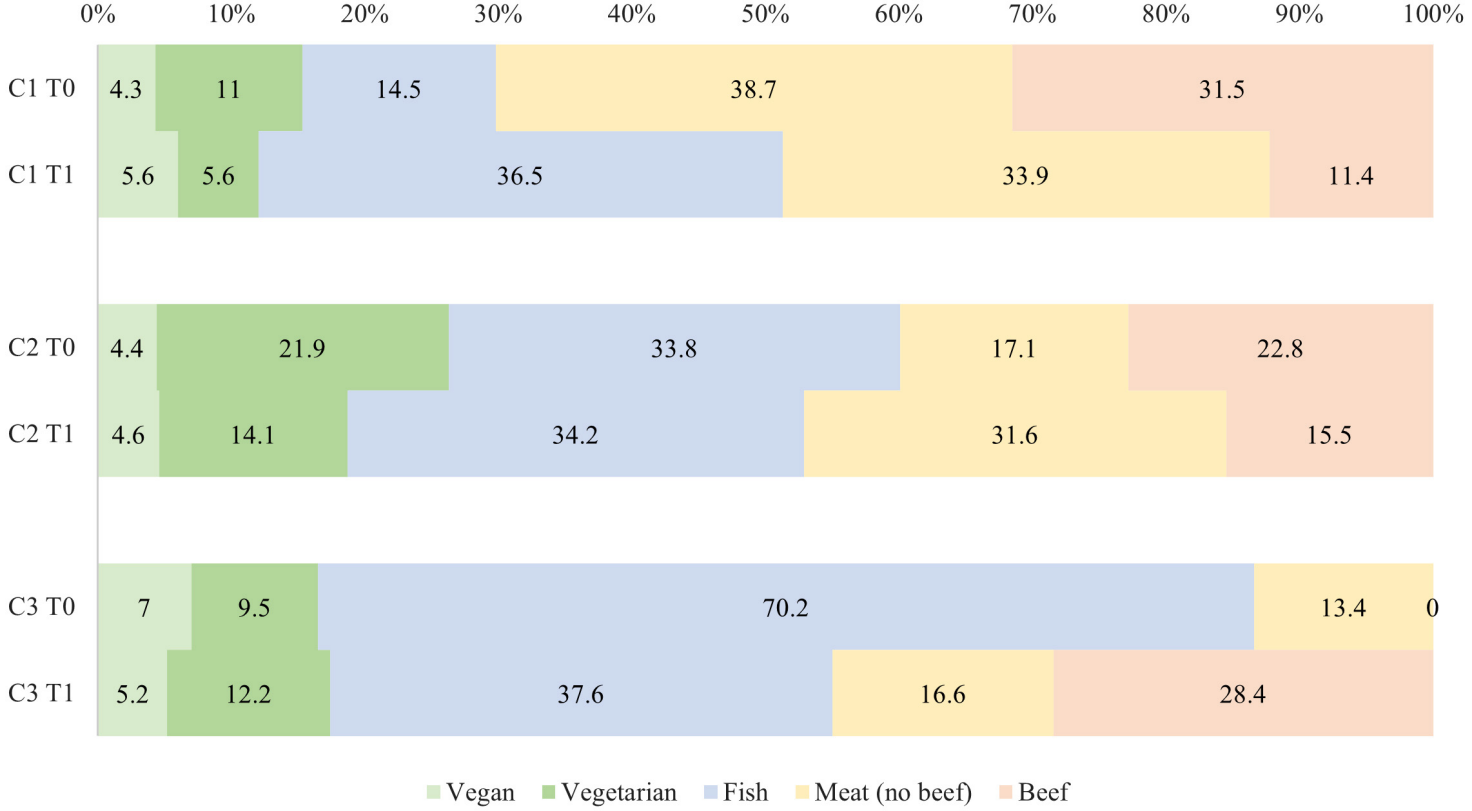
Categorization of trays in the two assessments (T0 vs. T1).

Figure 2 shows the categorization of trays in the pre- and post-intervention phases. A significant association between the distribution of tray categories and assessment period (T0-T1) was found for all three canteens (*p* < 0.001). In C1, during the post-intervention phase, trays categorized as fish increased (14.5% at T0 vs. 36.5% at T1), while those categorized as meat without beef (38.7% at T0 vs. 33.9% at T1) and beef (31.5% at T0 vs. 11.4% at T1) decreased. In C2, there was a significant increase in trays categorized as meat without beef (17.1% at T0 vs. 31.6% at T1) and a decrease in those with beef (28.8% at T0 vs. 15.5% at T1). In C3, there was a significant decrease in fish trays (70.2% at T0 vs. 37.6% at T1) and an increase in beef trays (0% at T0 vs. 28.4% at T1). Notably, the finding in C3 should be interpreted in light of the differences in menu composition between the two assessment periods. Beef-based dishes were not available during the T0 observation week but were introduced during the T1 assessment, suggesting that the increase in beef-containing trays may partly reflect changes in food availability rather than behavioral responses to the intervention. The complete results are available in Supplementary Table 3.

The analysis also revealed a significant association between the type of second course and the period of assessment (T0-T1) (*p* < 0.001). In C1, meat-based second courses (excluding beef) increased significantly (21.7% at T0 vs. 31.1% at T1), while those with beef decreased (22.4% at T0 vs. 10.2% at T1). In C2, fish second courses increased (8.8% at T0 vs. 22.1% at T1), while meat (excluding and including beef) decreased, but not significantly. In C3, meat second courses excluding beef decreased (80.4% at T0 vs. 37.6% at T1) and beef main courses increased (0% at T0 vs. 27.3% at T1). The complete results are presented in Supplementary Table 4.

As shown in Table 3, there was no significant association between fruit consumption and assessment period (T0-T1) in any of the three canteens. However, there is a significant association between side dishes and assessment time, in C1 and C3 (Table 3). Furthermore, in C1, where salad was switched to buffet distribution, 39.9% of users took salad at T1, compared to 23.3% at T0 (*p* < 0.001). More salad choices at T1 compared to T0 were also observed in C3, and at the threshold of significance at C2.

## 4 Discussion

This study provided an opportunity to evaluate the effectiveness of educational and environmental interventions in guiding users' choices toward healthier and more sustainable habits in three northern Italian hospital canteens. The intervention varied slightly according to the spaces and logistical possibilities of each canteen. Specifically, in C1, educational posters, fruit, pulses and salad self-service, and posters with descriptive norm messages were implemented; in C2, educational posters, a reversal of the flow of dish choices starting from the side dish, and posters with descriptive norm messages were implemented; and in C3, educational posters (excluding the suggested tray composition due to catering management limitations) and posters with descriptive norm messages were implemented.

C1, the largest canteen in terms of daily user rate and managed by an external catering service, had the macronutrient profile closest to the Italian reference values (29). Nevertheless, users' choices showed the worst environmental profile, probably due to the high selection of beef-containing foods and larger meals (i.e., highest median meal energy). In contrast, C3, which has the smallest user rate (and therefore the smallest sample size) and is the only canteen managed internally, showed considerable variability in results between T0 and T1. However, C3 represents, however, the canteen with the environmental profile and fiber intake closest to the reference ranges, but only at T0. Given the type of management and the resulting high variability in food offerings, only an extended observation period could provide truly representative data for this canteen. Finally, C2 is the canteen with the macronutrient profile that differs most from the Italian reference values at both assessment points. However, at T1, it has a median value for environmental indicators within the reference ranges, which may reflect the lower energy content of the meals.

### 4.1 Nutritional assessment

According to the guidelines (30), the energy content of a lunch meal suitable for a 2,000 kcal diet could be set between 700 and 800 kcal. At T0, the energy intake from the meal was below 700 kcal in C3 and above 800 kcal in C2, while at T1, the values were within the 700-800 kcal range in both canteens. However, in C1, the median energy value remained virtually unchanged and slightly above the identified range, indicating more caloric meals despite the intervention. As observed in the first assessment in 2022 (21), at T1, meals in all three canteens tended to be high in lipids. In C1 and C3, the contribution of lipids to energy intake was high but within the reference range (20%−35%E) at both time points (29), while in C2 it was higher, with a median of 40%E at T0 and 39%E at T1. Consequently, in C2, the contribution of carbohydrates to energy was below the lower limit of the reference range (45%−60%E) (29), with a median of 39%E at both time points. This observation confirms previous findings in studies evaluating workplace canteen settings, where meals often have an unbalanced nutritional profile, with high energy and fat content, which in turn is reflected in less healthy food choices by users (17, 31). Overall, our results suggest that the combined educational and environmental intervention was not sufficient to substantially modify macronutrient distribution. However, beyond energy and the total amounts of proteins, lipids, and carbohydrates, other nutritional components represent important targets for improving nutritional quality of the meals, particularly in collective catering settings, where reformulation strategies and changes in food preparation practices may contribute to healthier dietary patterns. Indeed, in terms of lipid quality, saturated fatty acid (SFA) intake remained within the recommended threshold of less than 10% of total energy across all canteens (29). Applying the thresholds of the Regulation (EU) No 1169/2011 EU Annex XIII, part B (32), one meal in median at T1 provides from 32 to 36% of the daily saturated fatty acids intake (20 g/day). Moreover, a statistically significant reduction in the energy contribution of SFAs was observed post-intervention across all three canteens. In C1 and C2, this decline coincided with a reduced selection of beef and cheese. In C3, the reduction in SFA contribution reflects a favorable shift in fatty acid composition, driven by a significant increase in the intake in grams of unsaturated fatty acids (MUFA and PUFA). Similarly, the intake of sugars at T1 in the three canteens (from 9% to 11% of total energy intake) falls within the recommended range of 15% (29). Applying the thresholds of the Regulation (EU) No 1169/2011 EU Annex XIII, part B (32), one meal in median at T1 provides from 7 to 13% of the reference daily sugar intake (90 g/day). It must be noted that this sugar intake primarily derives from choices regarding desserts (yogurt or pre-packed puddings) and fruit. Therefore, the reduction in the intake in grams of sugars in C2 may be explained by a lower choice of desserts and smaller portions of fruits then at T0, since fruit choice itself was not significantly affected by the intervention. Another important component to consider in collective catering settings, given its relevance to cardiovascular disease prevention, is sodium. However, it was not possible to estimate sodium intake because salt was not clearly quantified in the recipes provided by the canteen staff, and users could add salt at their discretion in the canteen hall.

Regarding fiber content of the meal, at T1, it decreased compared to T0 in C2 and increased in C1 and C3; in these last two the value falls within the reference range for adults (12.6–16.7 g/1,000 kcal) (29). One factor that may have increased fiber intake in C1 after the intervention is the partial change in service modality. At T0, users did not have autonomy in selecting fruit and vegetables, whereas at T1, pulses, salads, and fruits were offered as a buffet (notably, users often took portions of fruit, vegetables, and pulses that exceeded those allowed by canteen regulations) (21). Consistently, after the intervention, the presence of salad rose from 23.3% to 39.9%, while side dishes increased from 66.5% to 71.6%. This suggests that the combined effect of the posters raising awareness of healthy food choices and the presence of the buffet nudging choices positively impacted the selection of vegetables (33). On the other hand, the lack of improvement in fiber intake observed in C2 warrants further consideration. In this canteen, where the percentage of trays containing fruit and side dish remained unchanged and salads choice increased slightly, the decrease in fiber intake could be attributed to smaller portions compared to T0 and/or to the frequent presence of potatoes as side dishes. In fact, while Italian culinary tradition often includes potatoes as a side dish, these tubers cannot be considered vegetables, as their nutrient composition is more similar to that of cereal products, which are generally consumed as part of first courses or as bread and substitutes. Moreover, operational aspects of food service management may have influenced users' choices. For example, if vegetable dishes were sold out and not promptly replenished, users may have had fewer opportunities to select fiber-rich options. Therefore, the effectiveness of environmental interventions may depend not only on the introduction of healthier choices but also on their consistent availability throughout the service period.

The combined intervention of posters with the reversal of the flow of dish choice—starting from side dishes, salad, and fruit instead of first courses—introduced only in C2, appeared not to have had the desired effect. This contrasts with previous evidence showing that presenting healthier options first can increase their selection (10, 34, 35). The different outcome observed in this study may depend on contextual factors, such as established eating habits, limited meal time, and eating in groups, which may reduce users' responsiveness to environmental modifications.

The results suggest that communication strategies can encourage the choice of vegetables in some cases, but their effectiveness is influenced by the context in which they are implemented. In C1, where educational posters and descriptive norm messages were combined with a buffet service offering vegetables, pulses, and fruit, the intervention was associated with increased vegetable selection, supporting the hypothesis that information strategies are more effective when structural barriers to healthy choices are reduced (e.g., allowing independent selection of combinations and quantities of vegetables) (36). Conversely, the increase in vegetable selection observed in C3, where only educational intervention and social norms were implemented suggests that communication strategies may also be effective in smaller contexts where social cohesion and familiarity among users may strengthen peer influence (34).

Individual and contextual factors such as established food preferences and socioeconomic and cultural barriers hinder the transition to more sustainable food choices (35, 37) that improve health and reduce the overall ecological footprint (35, 38). Therefore, interventions in food service settings should consider not only nutritional recommendations but also the availability, accessibility, and social context influencing food selection.

In conclusion, the intervention strategies evaluated in this study appeared effective in encouraging the selection of salads and side dishes, whereas they did not significantly modify fruit consumption and they did not change substantially the energy and macronutrient composition of the meal, with few exceptions. This difference could be explained by the fact that fruit and vegetables, although often considered together in nutritional recommendations, respond to different behavioral determinants. Vegetables are generally perceived as a structural component of the main meal (39), and their choice can be more easily influenced by messages promoting healthy meals. In contrast, fruit is frequently associated with optional consumption, such as dessert or snacks (40), and is more influenced by individual preferences and established habits (41). Furthermore, fruit may require greater behavioral effort (i.e., peelling, slicing, eating by hand) than ready-to-eat vegetables, reducing the effectiveness of purely educational interventions (41). These results suggest that improving fruit consumption in hospital canteens may require more targeted environmental interventions such as increasing visibility, variety, and ease of access to the product (e.g., pre-cut fruit), as highlighted in the systematic review by Metcalfe J et al. (42).

### 4.2 Sustainability assessment

It is noteworhty that in C1 and C2, the median carbon footprint decreased after the intervention (from 1,338 g CO_2_ eq./meal to 1,227 g CO_2_ eq./meal in C1 and from 1,094 g CO_2_ eq./meal to 966 g CO_2_ eq./meal in C2). However, in C3, the median carbon footprint increased (from 773 g CO_2_ eq./meal to 1,126 g CO_2_ eq./meal). In a previous study on pre-intervention assessment, researchers highlighted that dishes containing beef have the highest value of carbon footprint (21), as beef is the food with the greatest environmental impact (43). It is estimated that 100 g of beef corresponds to approximately 2.5 kg CO_2_ eq, which is more than double the upper limit of the reference range for one complete meal (43). In C1 and C2, after the intervention, the number of trays and second courses containing beef decreased, likely contributing to the reduction in carbon footprint from T0 to T1 in both canteens. This reduction is further supported by the decrease in the selection of cheese as a second course, which, being derived from beef, contributes significantly to the carbon footprint. The choice of cheese decreased from 12.4% to 7.2% in C1 and from 14.5% to 12.9% in C2. These findings are consistent with the hypothesis that environmental interventions, combined with descriptive norm messages and educational strategies, may have contributed to greater awareness and more informed food choices. In C3, however, the carbon footprint increased, despite a significant decrease in the number of second courses containing meat (from 80.4% to 37.6%). This result can be explained by the absence of beef at T0, whereas at T1, during the week of analysis, two beef options were introduced among the second courses and one among the first courses (35, 37, 38). Although reductions in carbon footprint were observed in C1 and C2 after the intervention, these findings cannot be interpreted as evidence of the intervention alone. Since menu composition was not identical between T0 and T1, changes in food availability likely contributed to the observed differences. The C3 results illustrate this issue well, as the introduction of beef dishes during the T1 observation week substantially influenced the environmental profile of users' choices. Therefore, the present findings should be interpreted as reflecting the combined effects of behavioral interventions and contemporaneous modifications in the food offer. In addition, the environmental indicators reported in this study should be interpreted considering the system boundaries adopted for the life cycle assessment (LCA). As post-marketing stages, including food preparation and transport, were not included in the SU-EATABLE LIFE database, the estimated carbon and water footprints are likely to underestimate the absolute environmental impacts of the meals. Consequently, the absolute CF and WF values reported in this study should be considered conservative estimates. However, because the same methodological approach was consistently applied, the relative comparisons between food choices, between the two assessment periods, and with the SU-EATABLE LIFE reference ranges remain informative. This limitation has been widely recognized in the LCA literature. Truncated system boundaries have been shown to affect the magnitude of absolute environmental estimates while generally preserving the validity of relative comparisons (44, 45). Overall, despite the methodological limits in the absolute estimations of environmental impacts, all canteens showed high carbon and water footprint values both at T0 and T1. These findings suggest that behavioral interventions alone are unlikely to achieve substantial reductions in the environmental impact of meals unless accompanied by structural modifications to food procurement and menu planning. Therefore, alongside nudging interventions, hospital catering services should progressively align food purchasing policies with higher sustainability targets by reducing the routine availability of high-impact foods, such as meat (particularly beef) and cheese, and increasing the attractiveness of plant-based options. Behavioral nudges should therefore complement, rather than replace, structural interventions in the food environment. This approach may also help address users' perceptions that making healthy choices is difficult because of the limited availability and quality of healthy options. (46).

### 4.3 Strengths and limitations

The main strength of this study is the analysis of more than 2,850 actual meal choices across multiple hospital canteens, with a comparison of choices before and after combined interventions focused on educational and environmental prompts to identify valuable and suitable interventions in specific contexts. The results contribute to expanding the available data on the most effective types of intervention in collective catering for adults. As reported in the previous pre-intervention study (21), the evaluation of trays using photographic documentation enabled the identification of any non-standardized portions, allowing for a more accurate estimate of nutritional and sustainability variables. Additionally, no recipes from the literature were used; information on portions and preparation was provided directly by the staff of the individual canteens. The main limitation of this study is the lack of complete menu standardization between T0 and T1. Although observations were conducted during the same season to minimize seasonal variability, differences in the dishes available during the two assessment weeks may have influenced users‘ selections independently of the intervention. In C2, there was a rotational menu that changed weekly over 8 weeks; in C3, the menu was extremely variable, as it was an internally managed canteen without a pre-decided menu; while in C1, there were only two menu options per season that alternated. Consequently, the observed pre-post differences cannot be interpreted as a causal estimate of intervention effectiveness but rather as the result of the interaction between behavioral strategies and changes in food availability. This limitation is particularly relevant for C3, where the introduction of beef dishes during T1 substantially influenced the environmental indicators of food choices. Moreover, regarding nutritional data, it is necessary to consider that the analysis is limited to the lunch meal and therefore does not allow determination of whether any nutritional imbalance found in this meal is also reflected in the users' overall diet. Other limitations are reported in the pre-intervention study (21), such as: choices varied depending on the time (some dishes ran out earlier), the evaluation of trays was only visual and therefore estimated, and the analysis of CF and WF was based on an environmental database derived from the literature, which requires continuous updates, and did not include data on the post-marketing stages of the food supply chain (e.g., emissions from the transportation and kitchen preparation phases). Finally, it was not possible to implement a uniform intervention for all canteens, as it was tailored to each individual setting; furthermore, the single-arm pre-post design and the absence of a control group limit the ability to attribute the observed changes exclusively to the intervention, as external factors and unmeasured confounders may have influenced the results. Although the pre- and post-intervention samples showed similar characteristics, they were not statistically comparable; therefore, the findings should be interpreted as preliminary evidence of changes following the intervention rather than as definitive evidence of causal effectiveness.

### 4.4 Key findings, implications, and future research directions

Even after the intervention, the dishes chosen by users tended to be high in lipids and low in carbohydrates in terms of their contribution to energy intake, compared to the Italian dietary reference values. The different dishes offered in the two observation periods did not allow a robust comparison before and after the intervention in each canteen. In the larger, externally managed canteen (C1), the presence of salad, fruit and pulses in buffet style, along with descriptive norm messages and educational posters may have contributed to a higher fiber intake after the intervention. However, in the small externally managed canteen (C2) the unbalanced macronutrient profile, and the decrease in fiber content from T0 to T1, did not align with the expected increase in vegetable or fruit intake, suggesting a lack of effectiveness of the communicative elements combined with the reverse flow of dish choice starting from vegetables and fruit. From a sustainability perspective, the presence or absence of dishes containing beef within the food offer (observed in C3) was confirmed as a crucial variable in determining the carbon footprint of users' choices. The present findings reinforce that improving users' choices requires not only educational and behavioral approaches but also structural changes in institutional food systems. Procurement policies, menu planning and food availability should be aligned with nutritional and decarbonization targets to maximize the effectiveness of behavioral interventions. Indeed, canteens play a key role in providing options that meet nutritional and sustainability criteria. Moreover, future studies should also investigate the perception of the visual appeal and palatability of healthier food choices, as healthier and more sustainable options should not only be available but also desirable for users to select.

Although only modest results were obtained through the intervention based on food choices, hospital canteens remain favorable settings for promoting educational initiatives, thanks to the high volume of meals served and the social and cultural role of hospitals and healthcare workers in promoting healthy and sustainable lifestyles. In this context, encouraging a balanced diet not only supports the prevention of chronic diseases and can help reduce indirect healthcare costs in the long term, but also reflects a broader organizational vision that considers employee health a priority, promoting a healthy and cohesive working community.

## Data Availability

The raw data supporting the conclusions of this article will be made available by the authors, without undue reservation.
